# Haptic awareness changes when lying down

**DOI:** 10.1038/s41598-021-92192-1

**Published:** 2021-06-29

**Authors:** Kaian Unwalla, Michelle L. Cadieux, David I. Shore

**Affiliations:** 1 Department of Psychology, Neuroscience and Behaviour, McMaster University, 1280 Main Street West, Hamilton, ON L8S 4K1 Canada; 2 Multisensory Perception Laboratory, a Division of the Multisensory Mind Inc., Hamilton, ON Canada

**Keywords:** Psychology, Human behaviour

## Abstract

Accurate localization of touch requires the integration of two reference frames—an internal (e.g., anatomical) and an external (e.g., spatial). Using a tactile temporal order judgement task with the hands crossed over the midline, we investigated the integration of these two reference frames. We manipulated the reliability of the visual and vestibular information, both of which contribute to the external reference frame. Visual information was manipulated between experiments (Experiment 1 was done with full vision and Experiment 2 was done while wearing a blindfold). Vestibular information was manipulated in both experiments by having the two groups of participants complete the task in both an upright posture and one where they were lying down on their side. Using a Bayesian hierarchical model, we estimated the perceptual weight applied to these reference frames. Lying participants on their side reduced the weight applied to the external reference frame and produced a smaller deficit; blindfolding resulted in similar reductions. These findings reinforce the importance of the visual system when weighting tactile reference frames, and highlight the importance of the vestibular system in this integration.

## Introduction

The location of sensations on the skin surface are coded in an internal reference frame—adjacent locations on the skin activate adjacent neural tissue in the somatosensory cortex. To interact with the objects causing these sensations, this internal, body-centric reference frame, is remapped to an external reference frame, most likely coded in the posterior parietal cortex^1^. To accurately localize a touch in space, both the posture of the body and its position in space must be considered since the limbs have multiple degrees of freedom. One posture—crossing the hands over the body midline—has provided great insight into the two reference frames used to localize a tactile stimulus in space. The present experiments examined the impact of altering visual and vestibular frames of reference during tactile localization.

The tactile temporal order judgment (TOJ) task provides an excellent index of the deficit that occurs when crossing the hands over the midline. This task asks participants to indicate which hand received the first of two vibrations, one presented to each hand^2–6^. This task is completed while the participants’ hands are uncrossed and when the hands are crossed over the midline. Consistently, accuracy is reduced when the hands are crossed compared to uncrossed. The integration model of this crossed-hands deficit^2,4^ assumes that tactile remapping occurs automatically, producing spatial coordinates for the touch. Responding to the tactile stimulus requires integrating the external, spatial, coordinate with the internal, skin-based, coordinate. The deficit observed when the hands are crossed arises because of differential weights placed on the two coordinates when determining the location of the touch. It is important to note that in a crossed-hands posture, these external coordinates point to the incorrect response. As a result, the integration model suggests that a larger weight placed on the external reference frame should lead to a larger crossed-hands deficit.

In line with the integration model, removing visual information leads to a smaller crossed-hands deficit^7^. The use of a blindfold likely decreases the reliability of the external reference frame by impeding the ability to visually locate the hands in external space. Similarly, late-blind individuals also show a smaller crossed-hands deficit with the magnitude of the deficit being similar to that observed among blindfolded participants^8^. No crossed-hands deficit is measured in congenitally blind participants, with high accuracy observed in both crossed and uncrossed postures^8,9^. Together, these results highlight the role of vision in establishing the reliability of the external reference frame.

Similarly, we predict that the reliability of the external reference frame may be reduced by manipulating the perceived direction of upright (i.e., the subjective vertical). Multiple cues contribute to the subjective vertical, such as the orientation of mono-oriented objects in the visual environment, the impact of gravity on the vestibular organs, and the pressure felt from surfaces underneath our body^10,11^. Lying down misaligns these sources of information with respect to the direction of upright and reduces the reliability of the subjective vertical.

Evidence for this reduced reliability can be derived by comparing the subjective visual vertical (SVV) to the perceptual upright (PU)^10^. During the SVV task, participants indicate when a line is oriented with the direction of gravity: “what direction will a ball fall when dropped”. For the PU task, participants are asked to indicate whether a visually presented letter is a ‘p’ or a ‘d’. The reported letter provides an indirect measure of the participants’ perception of upright, as both are identical characters, except rotated 180 degrees. These tasks were differentially affected by lying down on one side. When sitting upright, both the SVV and the PU produce similar responses: all cues (body, visual, and gravity) indicate the same direction for upright. Lying down produces different responses in these two tasks: the SVV remains aligned with gravity while the PU aligns with the body, revealing two different perceptual directions of upright.

These conflicting signals, when lying down, should degrade the overall reliability of determining which way is up. Altering the reliability should impact the relative weights placed on the internal and external reference frame. Since body-based cues for upright will be misaligned with visual and gravitational cues (obtained through the vestibular system), the overall reliability of the external reference frame should be reduced, leading to a lower external weight, and a smaller crossed-hands deficit. However, it is also possible that the reliability of the subjective vertical does not influence the external reference frame, in which case, we would expect to see a similar magnitude of deficit in the crossed-hands posture when lying down compared to when sitting upright.

In the present studies, participants completed a tactile TOJ task by indicating which hand received the first vibration with their hands crossed and uncrossed. They completed this task while sitting upright or lying on their left side. Experiment 1 allowed participants to see their hands and the room around them. Experiment 2 removed the misaligned visual information by having new participants perform the same task wearing a blindfold. The key finding relates to the relative size of the crossed-hands deficit when sitting upright and lying down; the impact of blindfolding should replicate previous findings demonstrating a reduced deficit^7^.

## Methods

### Participants

Participants reported normal or corrected-to-normal vision and were naïve to the purpose of the experiment. All participants provided written informed consent prior to participation, and were remunerated with one course credit. All procedures were approved by and conformed to the relevant guidelines and regulations of the McMaster Research Ethics Board, and complied with the tri-council statement on ethics (Canada). For Experiment 1, twenty participants (10 males; 14 right-handed) with an average age of 18.4 years were recruited from McMaster University, using an online recruitment tool. For Experiment 2, twenty (10 males; 16 right-handed) new participants, with an average age of 22.5 years were recruited using the same recruitment and screening procedures. Sample sizes were determined based on the conventional number of participants used in past tactile TOJ studies. All participant data were included in the presented analysis for both experiments.

### Apparatus and stimuli

**Figure 1:**
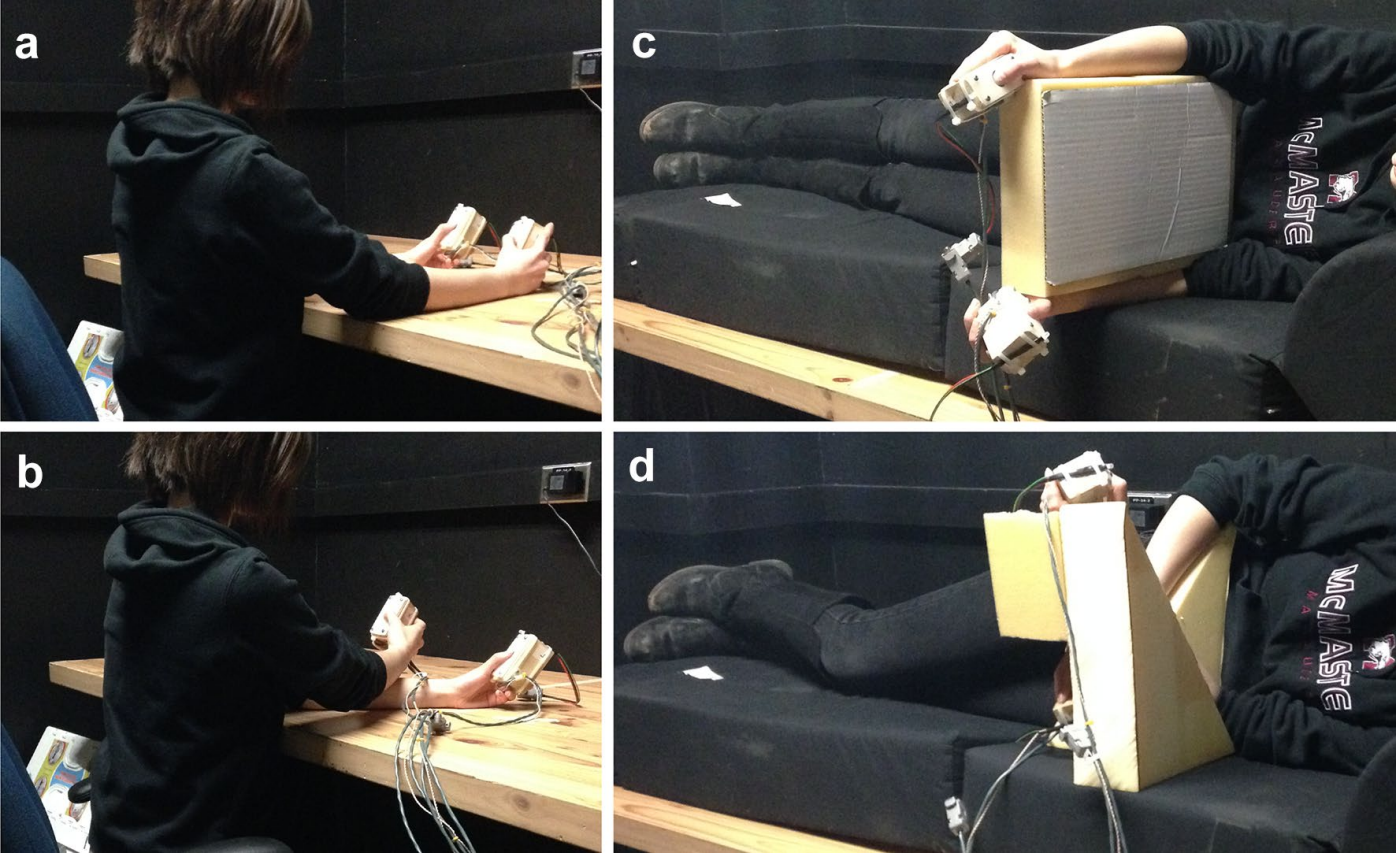
Hand and body postures used for Experiments 1 and 2. (**a**) Participant sitting upright with hands uncrossed, (**b**) participant sitting upright with hands crossed, (**c**) participant lying on left side with hands uncrossed, and (**d**) participant lying on left side with hands crossed.

Participants were seated at, or lying down on a table (73.7 cm in height). When lying down a soft foam mattress was placed on the table for comfort. They held two wooden boxes, separated by 18 cm with their thumbs in contact with the tactile stimulators. In the lying down position the participants’ hands were supported by foam triangles to mimic the hand position while upright (Fig. 1). The tactile stimulator (100 Ohm Oticon-A bone-conduction vibrator, measuring 1.6 cm in width and 2.4 cm in length) was placed on top of the response buttons, and the entire apparatus was enclosed in a small wooden box with a Plexiglas top. A 2 cm diameter hole was cut into the Plexiglas for participants to place their thumbs and push down on the vibrator to make a response. The vibrators were driven by an amplified 250 Hz sine wave that was suprathreshold and identical for all participants. All stimulation was controlled by a set of reed-relays connected to the parallel port of a DELL Dimension 8250, running Windows XP software. Matlab was used to deliver the stimulation and collect the participants’ responses. Participants wore earbud headphones playing white noise to mask the sounds produced by the tactile vibrators.

Experiment 2 used the same apparatus, but additionally had a pair of swimming goggles with the lenses painted black as a blindfold for participants.

### Procedure

Participants first completed two practice blocks, each with 16 trials. The first block of practice trials was completed with their hands uncrossed, and the next with their hands crossed over the midline. Hands were crossed with their right hand on top. The practice trials were completed either upright or lying down, depending on which posture the participant completed first. The experimenter was in the room during the practice trials to provide feedback and answer questions. Participants then completed 12 experimental blocks, each with 64 trials. The number of trials was chosen in order for the experiment to be completed within 1-h and to ensure the participant did not get uncomfortable or tired. The experimenter would start the block and leave the room, check on the participant after each block and start the next block. The experimental blocks were broken down into four sets of three blocks each. The first six blocks were completed either sitting upright or lying on their side, and the subsequent six were completed in the alternate body posture. Within each body posture, three consecutive blocks were completed with their hands uncrossed, and the other three with their hands crossed. The starting hand posture and body posture were counterbalanced across participants.

Each trial began 800 ms after the participant’s previous response. Each trial consisted of two 20 ms vibrations, one to each thumb, separated by one of four fixed stimulus onset asynchronies (SOAs): ± 400, ± 200, ± 100, ± 50 ms, where negative SOAs indicate the vibration was to the left hand first. This resulted in 24 trials for each body posture, hand posture, and SOA. After the second vibration occurred, participants responded by pressing down on the vibrator held in the hand that received the first vibration. If no response occurred within three and a half seconds of the second vibration, the trial timed out. To alert the participant that they missed a trial, both buttons vibrated three times. To move on, participants pressed and released both buttons. Time out trials and trials with premature responses (i.e., responses made less than 100 ms after the second vibration) were removed from all analyses. This resulted in the removal of 16 trials across 9 participants in Experiment 1, and 32 trials across 10 participants in Experiment 2.

Experiment 2 followed the identical procedure, except all participants were blindfolded for the entire experiment.

### Analysis

The magnitude of the crossed-hands deficit was evaluated using the proportion correct difference (PCD) score^3,12^. The difference in accuracy between the crossed and uncrossed postures were summed across SOAs. To determine whether the size of the deficit differed based on body posture and visual information, a 2 × 2 analysis of variance (ANOVA) was conducted on the PCD scores with body posture (upright vs. lying down) as a within-subject factor and visual information (blindfold vs. no blindfold) as a between-subject factor. One sample *t*-tests, comparing the PCD score to 0 were conducted to evaluate the presence of a crossed-hands deficit. Given that these were hypothesis driven tests, we did not correct for multiple comparisons. For the ANOVA, effect-size was computed as eta-squared, and as Cohen’s d for the *t*-tests. All significance tests were two-sided and used an alpha level of 0.05.

Reference frame weights were calculated using the equations outlined by^2,23^. These equations were implemented using a modified version of their hierarchical model. An internal and external weight (*ω*) pair were used to generate the slope (*θ*) of the logistic function; the probability of a right-first response as a function of SOA (t) for both crossed and uncrossed postures (Eq. 1). For the uncrossed posture the internal and external reference frames provide congruent information, so *θ* is the sum of the internal and external weights. In the crossed posture the reference frames conflict, so *θ* is the difference between the weights.

1 $$p\left( t \right) = ~\frac{1}{{1 + e^{{ - \theta t}} }}$$

where

$$\begin{aligned} & \theta _{{uncrossed}} = \omega _{{internal}} + \omega _{{external}} \\ & \theta _{{crossed}} = \omega _{{internal}} - \omega _{{external}} \\ \end{aligned}$$

Each experiment (blindfold vs. no blindfold) was modelled separately. For each experiment, a Markov Chain Monte Carlo (MCMC) simulation using a Metropolis–Hastings sampling algorithm, simulated using R studio, was used to provide an estimate of the population internal weight, external weight, and the standard deviation associated with the weights (see^2,23^). These parameters were approximated by truncated Gaussian distributions (limits = 0, ∞), with unknown means and standard deviations. Strictly positive, uniform hyperpriors were applied to all population parameters. The priors on the individual participants’ weights were constrained by the population distribution.

On each trial of the MCMC simulation, a new hypothesis was generated. Each hypothesis consisted of 46 parameters: 6 population-level parameters and 40 participant-level parameters. The 6 population parameters were: the mean internal and external weights (*μ*_*internal*_ and *μ*_*external*_) and standard deviations (*σ*_*internal*_, *σ*_*external*_) in the upright posture, and an internal and external weight task context parameter (*δ*_*internal*_ and *δ*_*external*_). The task context parameter determines the magnitude of the effect the body posture manipulation has on the internal and external weight. A context parameter less than 1 indicates the weight was reduced when lying down; a context parameter greater than 1 indicates that the weight increased when lying down; while a context parameter equal to 1 would indicate no change in the weights when lying down. As is customary in MCMC modelling, the manipulation (lying down) was assumed to affect all participants equally; as such, the weights while lying down were calculated by multiplying each participants’ upright weights by the population task context parameters. For example, if the task context parameter was 2, then the weights while lying down would be twice as large as the weights while upright. To be clear, the posterior distributions of two parameters were approximated for each individual participant: an internal and external weight (*ω*_*internal*_ and *ω*_*external*_) for the upright condition; the lying down condition used these same weights and multiplied them by the population context parameters (*δ*_*internal*_ and *δ*_*external*_). One hypothesis generated four psychometric curves for each participant—two for each body posture and two for each hand posture.

2 $$p\left( {H|D} \right) \propto ~\mathop \prod \limits_{i}^{N} p{\text{(}}d_{i} |\omega _{{int~i}} ,\omega _{{ext~i}} )_{i} \mathop \prod \limits_{i}^{N} p(\omega _{{int~i}} ,\omega _{{ext~i}} |\mu _{{int,}} \mu _{{ext}} ,\delta_{{int}}, \delta_{{ext}} ,\sigma _{{int}} ,\sigma _{{ext}} {\text{)}}p\left( {\mu _{{int,}} \mu _{{ext}} ,\delta_{{int}}, \delta_{{ext}} ,\sigma _{{int}} ,\sigma _{{ext}} } \right)$$

$$\begin{aligned} &{\text{where}}: \end{aligned}$$

$$ H = \left\{ {\omega _{{int~i}} ,\omega _{{ext~i}} ,~\mu _{{int,}} \mu _{{ext}} ,\delta_{{int}}, \delta_{{ext}} ,\sigma _{{int}} ,\sigma _{{ext}} } \right\}$$

$$ i = 1.$$

$$ N = {\text{number }}\;{\text{of}}\;{\text{ participants}}\;{\text{ in }}\;{\text{the}}\;{\text{ experiment}} $$

3 $$\log \left( {p\left( {d_{i} |\omega _{{int~i}} ,\omega _{{ext~i}} } \right)} \right) = \mathop \sum \limits_{{\text{t}}} r_{{t~i}} \cdot \log \left( {p_{i} \left( t \right)} \right) + l_{{t~i}} \cdot \log \left( {1 - p_{i} \left( t \right)} \right)$$

$$\begin{aligned} &{\text{where}}: \end{aligned}$$

p(t) is defined in Eq. (1);

$$d_i \,\text{=\,The data from participant i; }$$

$$\begin{aligned} \omega _{{int~i}} ,\omega _{{ext~i}} \text{=\,the hypothesized internal and external weight values for each participant;}\end{aligned}$$

$${r_{t i}}\;\text{=\,number\;of\;right-first\;responses;} $$

$${l_{t i}}\,=\,\text{number of left-first responses (i.e., the number of trials-number of right first responses);} $$

$${t}\,\text{in}\,\rm \{{400,\,\pm\,200,\,\pm\,100,\,\pm\,50}\}.$$

Bayes’ formula was used to calculate the posterior probability of a hypothesis, H, given the data set (D) (Eq. 2). The probability of each participant’s data (d_i_) given the participant’s hypothesized weights were calculated using the binomial distribution at each SOA (Eq. 3), with proportion right-first responses p(t). This probability was then multiplied by the prior probability of the upright weights. The joint posterior, P(H|D), was then approximated using MCMC.

For each Experiment, five Markov chains with 250,000 samples were run, with the first 50,000 samples removed as the burn-in period. These values were chosen to ensure an appropriate convergence of the model. Experiment 1 had a convergence metric, $\hat{R}$, between 0.91 and 1.02, indicating that the chains had converged^13^. Experiment 2 showed similar convergence with $\hat{R}$ between 1.00 and 1.02. Random values for each of the 46 parameters were chosen as initial values for the chains. Future parameter values were chosen from a Gaussian distribution with a mean centered on the previous parameter value and proposal standard deviations of 0.26 for the weights, 0.23 for the population standard deviations, and 0.02 for the task context parameter. We selected these values to obtain acceptance rates between 20 and 35%^14^. In Experiment 1 all runs had an acceptance rate of 21%, and in Experiment 2 the acceptance rate was between 32 and 33%.

## Results

**Figure 2:**
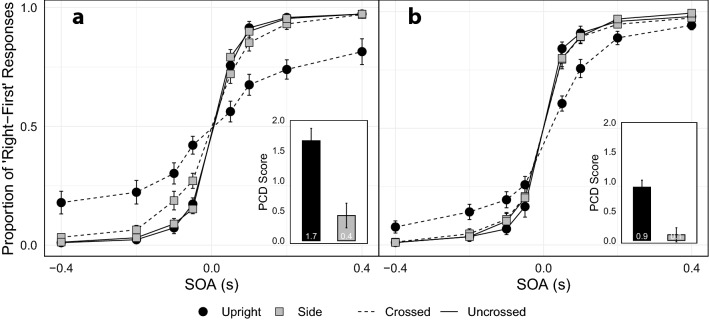
Overall proportion of right-first responses while wearing (**a**) no blindfold and (**b**) a blindfold. Participants indicated which hand was vibrated first, and the average proportion of ‘right-first’ responses was calculated at each SOA. Negative SOAs indicate the left hand received the vibration first. The average PCD score for each condition is represented in the bar graphs. Error bars represent standard error of the mean corrected for a within-subject design^15,16^.

A separate PCD score was calculated for each participant in both the upright and lying down postures. Data from Experiment 1 and Experiment 2 were submitted to the same ANOVA with body posture (upright vs. lying down) as a within-subject factor, and vision (no blindfold vs. blindfold) as a between-subject factor. Based on the PCD scores, lying down produced a smaller deficit (M = 0.26, SD = 0.59) compared to sitting upright (Fig. 2; M = 1.28, SD = 1.03; *F*_(1,38)_ = 37.37, *p* < 0.001, $\eta _{g}^{2}$ = 0.30). Blindfolding further reduced the deficit (M = 0.50, SD = 0.63) compared to intact vision (M = 1.04, SD = 1.18; F_(1,38)_ = 8.21, *p* = 0.007, $\eta _{g}^{2}$ = 0.11), replicating results using similar methods^7^. There was no significant interaction between body posture and vision (F_(1,38)_ = 1.70, *p* = 0.201, $\eta _{g}^{2}$ = 0.02). One-sample *t*-tests comparing the PCD score to 0 confirmed that the crossed-hands deficit was evident with intact vision (Upright: t_(19)_ = 6.16, p < 0.001, *d* = 1.38; Side: t_(19)_ = 2.37, p = 0.03, *d* = 0.53) and while blindfolded (Upright: t_(19)_ = 6.12, p < 0.001, *d* = 1.37; Side: t_(19)_ = 2.23, p = 0.03, *d* = 0.51); the size of the deficit when lying down and blindfolded was less than 5% the size of the deficit seen when upright with vision.

**Figure 3:**
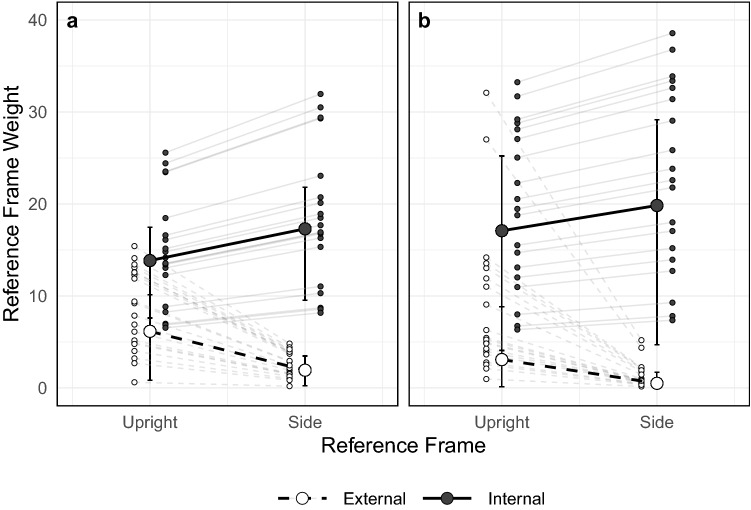
Overall population internal and external weights for each body condition based on whether participants were wearing (**a**) no blindfold or (**b**) a blindfold. Error bars represent 95% credible intervals calculated directly from the MCMC posterior distributions. The small circles represent individual participant weights.

Population estimates for the internal and external weights were derived using a Markov Chain Monte Carlo simulation, with the posterior probability of each hypothesis being calculated using Bayes’ formula. To determine whether the internal and external weights (Fig. 3) were affected by the manipulation of lying down, we calculated equal-tail 95% credible intervals on the task parameters. For both experiments, the external task parameter was less than 1 (Experiment 1: 0.31 [0.22, 0.41], Experiment 2: 0.16 [0.04, 0.30]), indicating a drastic reduction in the weight assigned to the external reference frame. The internal task parameter was larger than 1 (Experiment 1: 1.25 [1.16, 1.35], Experiment 2: 1.16 [1.08, 1.25]), indicating an increase in the weight placed on the internal reference frame. Based on the weight parameters observed, both manipulations resulted in large reductions to the external weight and a small increases in the internal weight.

## Discussion

The novel findings here concern the effect of lying down on the size of the crossed-hands deficit. Based on the modelling of these data, we conclude that this reduced deficit emerges primarily from reducing the weight to the external reference frame. This finding was replicated across the two experiments, with and without vision. When blindfolded (Experiment 2), we observed a further numerical decrease in the magnitude of the crossed-hands deficit. Modelling showed a concomitant decrease in the external reference frame weight. The smallest deficit was measured when blindfolding was combined with lying down suggesting that both manipulations contribute to the simulation of the external world in independent ways. It is important to note that the role of visual information was compared using a separate group of participants. As such additional studies are needed to confirm the combined influence of lying down and blindfolding.

The impact of blindfolding replicates previous findings^7^. In the absence of visual information, either through blindfolding^7^ or being congenitally blind^8,9^, a smaller crossed-hands deficit is observed. Our results extend these previous findings by showing that the decreased deficit is the result of less weight being placed on external information. This further supports the external coordinates for touch being strongly linked to visual information.

Further evidence for the involvement of vestibular information during tactile localization comes from the application of galvanic vestibular stimulation (GVS), which stimulates the vestibular nerve. When applied, participants are less accurate at localizing where a touch occurred on the hand^17^, and poorer at locating where their arm is in space^18^. Vestibular information also helps provide a sense of body ownership. Currently we accept that our sense of body ownership and posture are malleable and heavily influenced by vision. Consider the rubber hand illusion^19^ where seeing a rubber hand brushed while simultaneously feeling your own hand brushed induces a sense of ownership over the rubber hand. When GVS is applied during the rubber hand illusion a larger proprioceptive drift is observed in the direction of the rubber hand, indicating greater ownership over the rubber hand^20^.

It is generally accepted that the crossed-hands deficit is the result of a weighted integration of information from the internal and external reference frames. However, some recent studies have called into question the assumption that the conflict occurring during the integration process is the result of the external spatial location of the touch^21,22^. These studies have shown that the external location may not be required to locate the touch to the hand, and instead the conflict may stem from information related to the body side of the touch or the canonical body posture of the hand. While these studies do present an exciting avenue for future research, their ability to explain the results of many previous crossed-hands tactile TOJ studies remains to be seen (i.e., the role of task demands^7,9,23^, or the role of vision^7,8^).

Based on the present experiments it is impossible to fully disentangle the contributions of visual, vestibular, and body-based information to the weight placed on the external reference frame. Future studies could attempt to separate these influences, for example, by disrupting the vestibular system using galvanic vestibular stimulation, or using a microgravity environment^24^. Future work could expand these underlying assumptions about this reference frame, as this has implications on how we understand our body representation^25^.

## Data Availability

The data generated and analysed in this paper along with accompanying reaction time data are available from the authors on request.
